# Annual Report of the Longview, Ohio, Asylum

**Published:** 1861-06

**Authors:** 


ANNUAL REPORT OF THE BOARD OF TRUSTEES AND OFFICERS OF LONGVIEW ASYLUM, TO THE GOVERNOR OF THE STATE OF OHIO—For the year 1860. Columbus, Ohio.

Two neatly printed reports, of no special interest except to those directly concerned. They serve to show, however, the attention given to and interest in this branch of the healing art; and their statistical tables will be valuable to the future student or writer on this speciality. The Friend's Asylum is the first in this country, in which any marked improvement over the olden barbarities of the strait-jacket and solitary confinement in worse than prison-cells, was introduced in the treatment of Insanity; and its successful establishment marks a happy era in the history of the disease in this country. Dr. Worthington furnishes, in his report, nine statistical tables, based on the conditions, occupations, etc., of some 1,430 patients.

The Longview Asylum, in actual operation only since February or March, 1860, is probably the largest, best constructed, and best arranged Insane Asylum in the country ; the edifice, being of brick, six-hundred and twelve feet long, from three to five stories high, from forty-five to one hundred and seventy-six feet deep, exclusive of a long, low building continued back one hundred and seventy feet in the rear of the main building, and an infinitude of rotundas, chapels, wards, dining, reception, bath, bed, lumber, reading, library, servants', work, clothing, dead, and other rooms, of bowling-alleys and billiard-halls, air ducts, dust and clothes-chutes, boilers, flues, stories, wings and corridors, gymnasia, etc., etc., etc., before which the details of the *Great Eastern* sink into insignificance. Dr. Langdon, the Superintendent and Physician, will find abundant employ for his well-known skill, energy and ability, in the conduct of this Institution, the existence of which, to quote his own words, is indeed not only a strong proof of the highest type of civilization in any community which originates and sustains such, but while effecting the more direct and obvious good results for which it is designed, it cannot fail to have an indirectly beneficial influence upon the community, by keeping constantly before it a prominent instance of far-sighted benevolence, in which the question of expense has been kept entirely subordinate to that of relieving in the most effectual manner, the affliction of a most unfortunate class.

